# Experimental Characterization and Calibration of a MEMS Electric Field Sensor Under DC Ionized Field Conditions

**DOI:** 10.3390/mi17030317

**Published:** 2026-03-03

**Authors:** Ren Ren, Bing Li, Chunrong Peng

**Affiliations:** State Key Laboratory of Transducer Technology, Aerospace Information Research Institute, Chinese Academy of Sciences, Beijing 100190, China; beyondlibing2008@126.com (B.L.); crpeng@mail.ie.ac.cn (C.P.)

**Keywords:** MEMS, electric field sensor, calibration method, DC ionized field

## Abstract

Accurate electric field measurement in high-voltage direct current (HVDC) environments is essential for power system monitoring. This study systematically investigates the output characteristics of a micro-electro-mechanical system (MEMS) electric field sensor under DC ionized field conditions. Using a controlled experimental platform capable of generating independent nominal electric fields and ion flows, the influence of ion current density on sensor sensitivity and offset was quantitatively analyzed. Experimental results reveal that ion flow leads to a progressive output drift and significant measurement deviations when using conventional electrostatic calibration. To address this issue, a joint calibration method incorporating ion current density is proposed. Validation experiments demonstrate that the proposed method significantly improves measurement accuracy, reducing the maximum relative error from 29.28% to approximately 5.07%. This work provides a reliable experimental basis and calibration methodology for utilizing MEMS electric field sensors in complex ionized DC environments.

## 1. Introduction

Electric field measurement plays a fundamental role in scientific research and engineering practice by providing direct information about charge distribution, field–matter interactions, and the operating states of electrostatic and electromagnetic environments. In meteorology, atmospheric electric fields are monitored to characterize charge accumulation and transport, supporting lightning warning and related atmospheric studies [1,2,3,4]. In power systems, mapping electric field strength around high-voltage equipment and transmission corridors is essential for condition assessment, insulation evaluation, and corona/flashover risk management [5,6,7]. In industrial scenarios, electric-field measurements are widely used for electrostatic control, non-contact inspection, and electromagnetic compatibility (EMC) testing [8,9,10], where field exposure and field distortion can critically affect reliability. Among these applications, accurate electric field measurement in the vicinity of HVDC transmission lines is particularly important for power-system inspection and real-time condition monitoring [11], since electric field signatures are closely linked to corona activity and abnormal operating states that may precede faults.

Electric field sensors can be broadly grouped into three major categories: field mill sensors [12], capacitive sensors [13], and optical sensors [11,14]. Field mills employ periodic modulation of an electrode to convert a static field into a measurable alternating signal, whereas capacitive sensors infer the field from induced charge or voltage on sensing electrodes. Optical sensors, typically based on electro-optic effects (e.g., the Pockels effect), provide a non-contact alternative in which the external field modulates optical phase or intensity for field reconstruction. This classification suggests that measurement accuracy is often limited by sensor–environment interaction and calibration robustness, especially in DC ionized fields where ion flow and surface charging can introduce additional bias.

In HVDC and atmospheric applications, conventional DC electric field meters are typically implemented as field mills or vibrating-plate instruments, which estimate field strength by periodically modulating the induced charge/current on sensing electrodes. Although these instruments are mature and widely adopted, their relatively large conductive structures and mechanical motion can introduce non-negligible field perturbation and may disturb charged-particle transport in ionized environments, where the spatial distribution of space charge and ion flow contributes to the total electric field itself. Under such DC ionized conditions, measurement accuracy is therefore constrained not only by sensor sensitivity but also by the robustness of calibration against ion-related effects (e.g., ion-current density and space-charge accumulation [15]), which can introduce additional bias and drift. These limitations motivate the development of MEMS-based electric field sensors [16,17,18,19,20]. Owing to their compact size, high level of integration, and the absence of macroscopic rotating parts, MEMS sensors can reduce installation constraints, mitigate field distortion caused by bulky structures, and avoid airflow-related disturbances to charged-particle transport. Consequently, MEMS electric field sensors have attracted increasing attention across multiple application domains, and their miniaturization potential makes them particularly attractive for field-sensitive and space-constrained deployment scenarios.

Under DC operating conditions, corona discharge on HVDC conductors can generate abundant space charge. The subsequent ion motion gives rise to an ion-flow-related field component that superposes with the background field set by the conductor voltage and the line–ground geometry. As a result, the total electric field in the vicinity of an HVDC line can deviate substantially from the nominal electrostatic field associated with the line voltage alone. Under severe corona conditions, the total electric field has been reported to reach two to three times the nominal field [8], leading to larger-than-expected field exposure and stronger spatial nonuniformity. This discrepancy between the nominal (voltage-determined) field and the total (space-charge-influenced) field provides the key motivation for modeling and quantifying ion-flow effects in HVDC environments. Existing research on HVDC ion-flow and space-charge modeling has been developed using several computational approaches. The flux-tracing approach [9,21] remains a common engineering strategy, in which the nominal field is computed first, and space-charge effects are then incorporated through flux-based corrections. Mesh-based methods, especially FEM, have also been widely used to directly solve coupled field–charge problems and to predict both electric-field distribution and ion-current density [22,23,24]. Complementary to these simulation studies, experimental investigations relevant to HVDC measurements have also been reported, including laboratory high-voltage platforms that emulate hybrid HVDC/HVAC electric field conditions for AC/DC component separation, electrostatic calibration, and long-term stability evaluation of dual-electrode electric field sensors [25]; prototype validation of 3D synthetic electric field vector measurement using electrostatic-field testing and parallel-plate-based field generation supported by FEM analysis and regression-based calibration [26]; and experimental mapping of space potential distribution in HVDC ion-flow fields using a suspended thin-wire probe with potential tuning, verified under scaled monopolar and bipolar configurations [27]. Overall, these studies have substantially advanced the simulation-based understanding of HVDC ionized fields and demonstrated experimental feasibility for high-voltage measurement; however, experimental studies are still limited for sensor calibration in DC ionized fields, particularly those that consider ion flow and surface charging and provide reliable calibration methods.

From a measurement and calibration perspective, HVDC environments with ionization should be treated as a coupled system of electric field and charged particles, rather than as a purely electrostatic problem. A key challenge is that the measured total electric field is the superposition of (i) the nominal component determined by the line voltage and the ground geometry and (ii) the component induced by corona-generated space charge. The space charge contribution can vary with operating conditions and environmental influences, thereby changing the relationship between the voltage determined nominal field and the measured total electric field. Moreover, when ion flow is present, sensor surfaces may collect and accumulate charge. This surface charge can distort the local field around the sensing structure and lead to output bias, drift, or apparent nonlinearity over time.

Therefore, calibration procedures established under purely electrostatic conditions may not remain valid when directly applied to ionized DC environments. Calibration and correction methods should explicitly consider ion flow effects and offsets caused by surface charge, ideally under controlled conditions where space charge is present and ion-related quantities can be measured. This enables repeatable characterization and calibration under HVDC-relevant conditions. However, the output behavior and calibration methodology of MEMS electric field sensors in ionized DC fields are still insufficiently established, particularly regarding the roles of ion flow density and surface charge accumulation.

To address these issues, this work systematically investigates the behavior of a MEMS electric field sensor in ionized DC fields and proposes a dedicated calibration method. The main contributions of this paper are as follows:

(1) The output characteristics of a MEMS electric field sensor under ionized DC field conditions are experimentally analyzed, with particular attention to ion flow density and surface charge effects.
(2) The influence of ion current density on sensor offset and sensitivity is quantitatively clarified.
(3) A joint calibration method considering nominal electric field, ion flow density, is proposed and experimentally validated.

## 2. MEMS Electric Field Sensor and Experimental Setup

### 2.1. DC Ionized Field Description

In DC ionized environments relevant to HVDC operation, corona discharge generates space charge that drifts under the electric field and forms an ion flow. Under quasi steady conditions, the coupled field–charge system can be described by the Poisson equation:

(1) $$\nabla \cdot E=\rho /\varepsilon_{0}$$

where *E* denotes the total electric field, ρ is the space charge density, and $\varepsilon_{0}\;$is the permittivity of free space.

The ion current density *J* is related to the space charge density and electric field by:

(2) J=ρμE

where μ is the ion mobility. Under steady conditions, the current continuity equation holds:

(3) ∇·J=0

The total electric field can be written as the superposition of the nominal (voltage-determined) field and the field induced by space charge,

(4) $$E=E_{nom}+E_{sc}$$

In controlled experimental systems, $E_{nom}$ can be determined from electrode geometry and applied voltage (e.g., $E_{nom}=V/d$ in an ideal parallel plate gap), while the ion current density can be independently measured. These relations provide the theoretical basis for analyzing sensor response and for developing calibration methods in DC ionized fields.

### 2.2. MEMS Electric Field Sensor

The MEMS electric field sensor used in this work was developed by our research group and is based on a resonant coplanar electrode structure. The sensor is designed for single-axis electric field measurement. The design and fabrication of this sensor have been reported in our previous work [28], where the sensor structure and performance characteristics are described in detail. Owing to its compact dimensions, the sensor introduces minimal distortion to the surrounding electric field. Unlike conventional field mill sensors, this MEMS sensor does not rely on mechanical rotation and therefore avoids airflow that could disturb the ion flow distribution.

**Figure 1:**
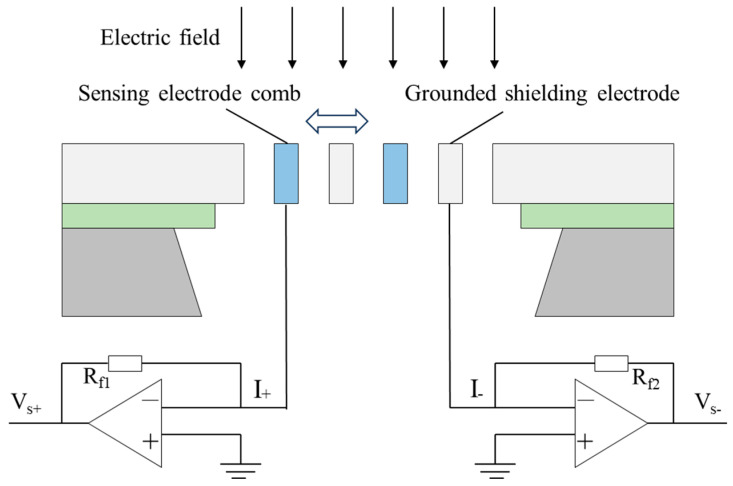
Schematic of the MEMS sensing microstructure and operating concept.

**Figure 2:**
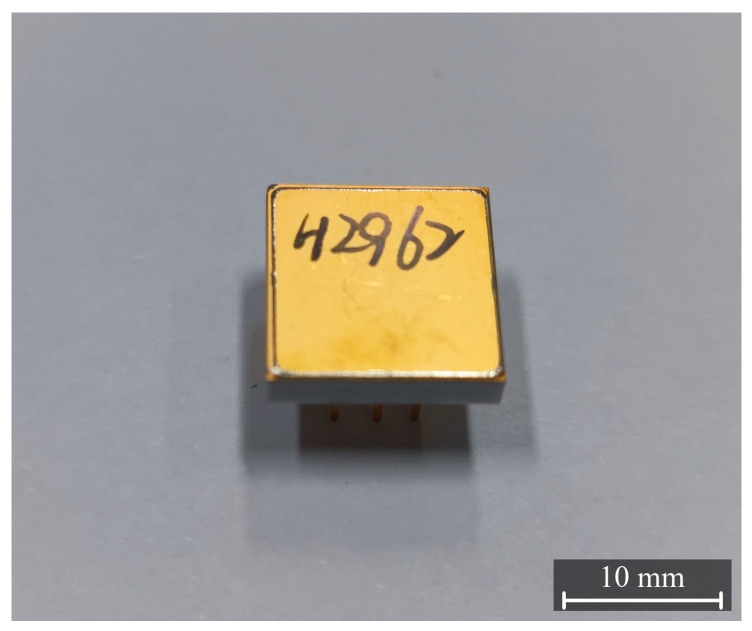
Photograph of the packaged MEMS electric field sensor module.

Figure 1 presents the sensing microstructure of the MEMS electric field sensor used in this study, while Figure 2 shows the packaged device. The packaged sensor module has an overall size of approximately 15 mm × 15 mm. The sensor adopts a coplanar electrode layout and a horizontal resonant architecture with electrostatic excitation. At the microstructure level, the device consists of differential sensing electrode combs and grounded shielding electrodes that form the sensing and modulation structure. When an external electric field is applied, charges are induced on the sensing electrodes. With a periodic excitation applied to the shielding electrodes, the effective coupling between the external field and the differential sensing electrodes is modulated, resulting in a cyclic variation in the induced charge. This modulation converts the DC electric field input into an alternating current electrical signal, whose amplitude is correlated with the external field strength. The induced signal is then processed through the readout chain, including amplification, demodulation, and voltage conversion, to provide the final output for analysis.

Given the micron-scale electrode features and gaps, the sensing structure is sensitive to environmental contamination; therefore, the MEMS chip is packaged into a protected module for practical high-voltage measurement scenarios. As illustrated in Figure 2, the package comprises a metallic lead frame that provides mechanical support and electrical connection, an insulating sidewall that ensures electrical isolation and forms the package cavity, and a metal sealing cap that protects the sensing chip. These metallic parts do not form a closed conductive enclosure and therefore do not shield the external electric field. When an external electric field is applied, induced charges are formed on the metal sealing cap, and the resulting electric field can still couple to the internal sensing electrode combs.

### 2.3. DC Ionized Field Generation System

**Figure 3:**
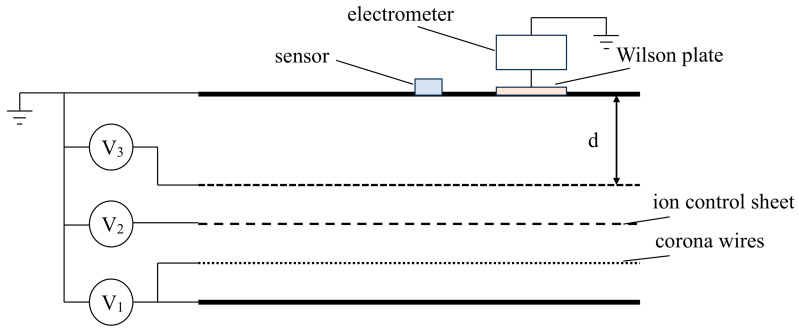
Schematic of the DC ionized field generation and test system.

**Figure 4:**
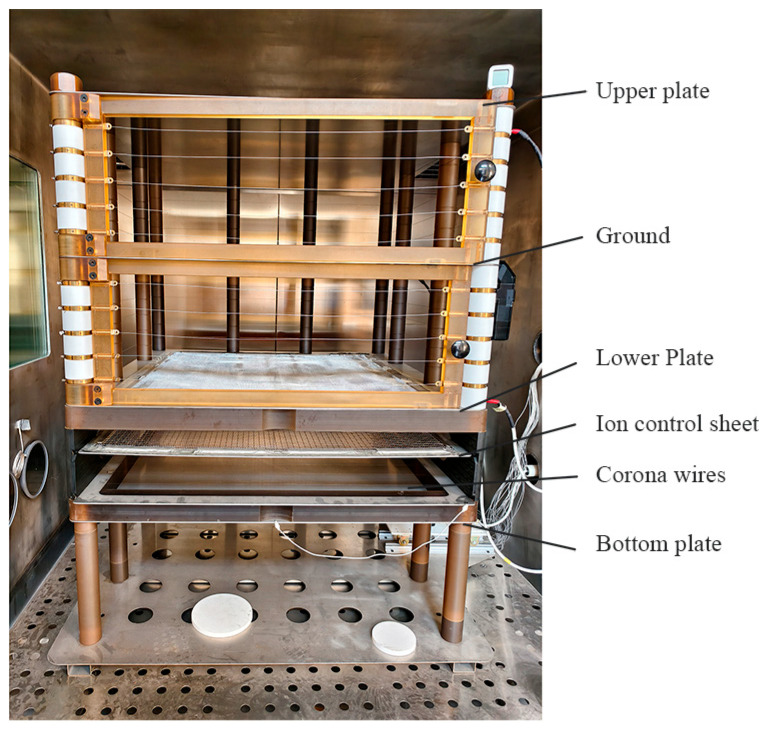
Photograph of the experimental setup.

Figure 3 presents a schematic diagram of the DC ionized field generation and calibration platform used in this work, while Figure 4 shows the corresponding photograph of the experimental setup. The two figures represent the same experimental system from schematic and physical perspectives. The system was developed in our laboratory and is designed to provide a controlled DC ion flow together with a controllable nominal electric field in a parallel plate test region. Overall, it consists of two functional parts: an ion generation and control section and a test area.

In the ion generation and control section, multiple corona wires are arranged above a metal plate. A DC high voltage V_1_ is applied to the corona wires together with the metal plate, so that corona discharge generates space charge and forms an upward ion flow. An ion control sheet biased at V_2_ is placed between the corona wire region and the parallel plate test region to regulate the amount of ions entering the test area and to improve ion flow uniformity. By adjusting V_2_, the ion current density in the test area can be tuned while keeping the physical layout of the test system unchanged.

The test area consists of an upper plate and a lower plate that form the parallel plate region. The upper plate is grounded and used for sensor mounting. A DC voltage V_3_ is applied to the lower plate to generate the nominal electric field, which is primarily determined by V_3_ and the plate spacing d (with $E_{nom}=V_{3}/d$ in an ideal gap). The MEMS electric field sensor and a Wilson plate are mounted on the grounded upper plate, and the sensing surface of the MEMS device is kept parallel to the inner surface of the grounded plate. The Wilson plate is connected to a high-precision Keithley 6517B electrometer (Keithley Instruments, Tektronix Inc., Solon, OH, USA) to measure the collected ion current.

It should be noted that when V_2_ is held constant, changing V_3_ may affect both the nominal electric field and the ion current density entering the parallel plate region. Therefore, V_3_ and V_2_ are adjusted according to the specific test condition to achieve the required combination of nominal field and ion flow level. This configuration enables controlled generation of DC ionized fields and provides measurements of nominal field setting and ion current density, supporting subsequent analysis of sensor output characteristics and calibration in DC ionized environments. All experiments were conducted under stable laboratory conditions at room temperature.

## 3. Output Characteristics of the MEMS Sensor

### 3.1. Standard Calibration Under Electrostatic Field

**Figure 5:**
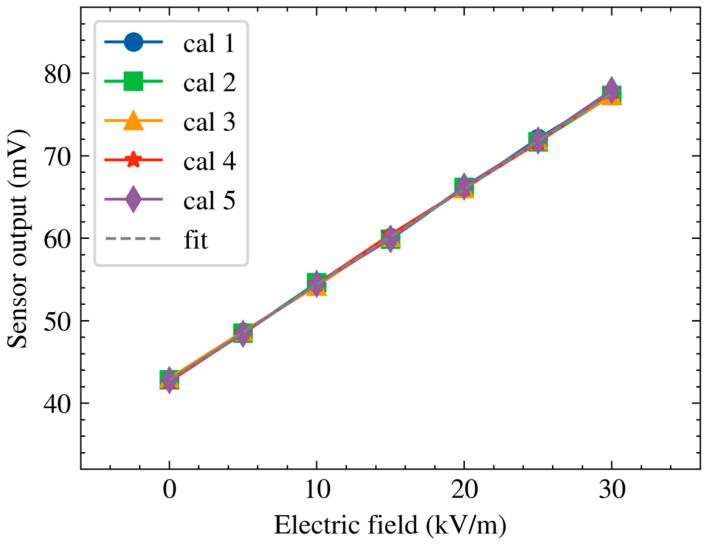
Electrostatic calibration curve of the MEMS electric field sensor.

As a reference baseline, the MEMS electric field sensor was first calibrated under a purely electrostatic field without ion flow. The nominal electric field in the parallel plate region was set by the applied voltage and plate spacing and was swept from 0 to 30 kV/m. Figure 5 shows the resulting calibration curves; the five calibration curves (Cal 1–Cal 5) correspond to repeated electrostatic calibration measurements performed under identical conditions without ion flow, demonstrating the repeatability and stability of the sensor response. The sensor output voltage increased linearly with the nominal electric field over the tested range. A linear fit yielded a sensitivity of approximately 1.16 mV/(kV/m), and the maximum fitting error was below 0.6%. These results confirm that the sensor provides accurate and repeatable electric field measurements under electrostatic conditions, which provides a baseline for evaluating output deviation and calibration validity in DC ionized fields.

### 3.2. Influence of Ion Current Density

After establishing the electrostatic calibration baseline in Section 3.1, the sensor response was examined in DC ionized fields.

**Figure 6:**
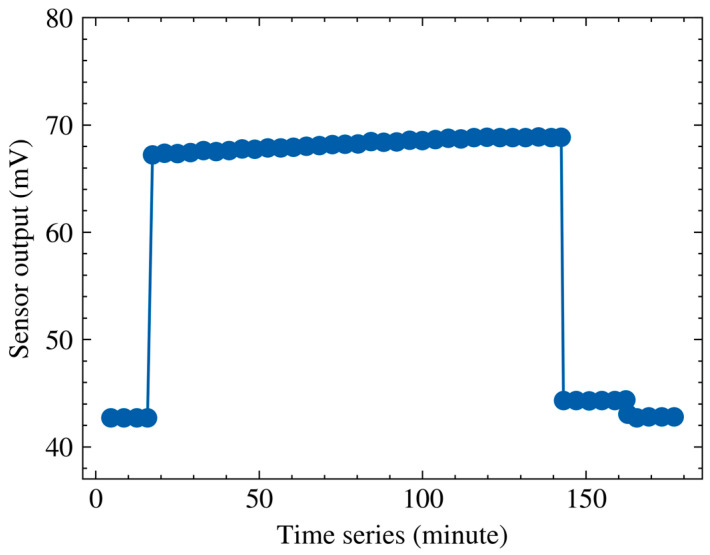
Sensor output drift under a fixed nominal electric field in a DC ionized field.

Figure 6 presents the evolution of the sensor output under a fixed nominal field in the ionized environment. Two abrupt changes in the output can be observed at approximately 15 min and 145 min, corresponding to the switching of the nominal electric field during the experiment. Between these transitions, a progressive drift is observed as the exposure proceeds. This drift is attributed to charge accumulation on sensor surfaces due to ion deposition. The accumulated charge can alter the local electric field distribution around the sensing structure and introduce an additional offset in the output. As a result, even when the nominal field is held constant, the output may gradually shift from its initial value.

**Figure 7:**
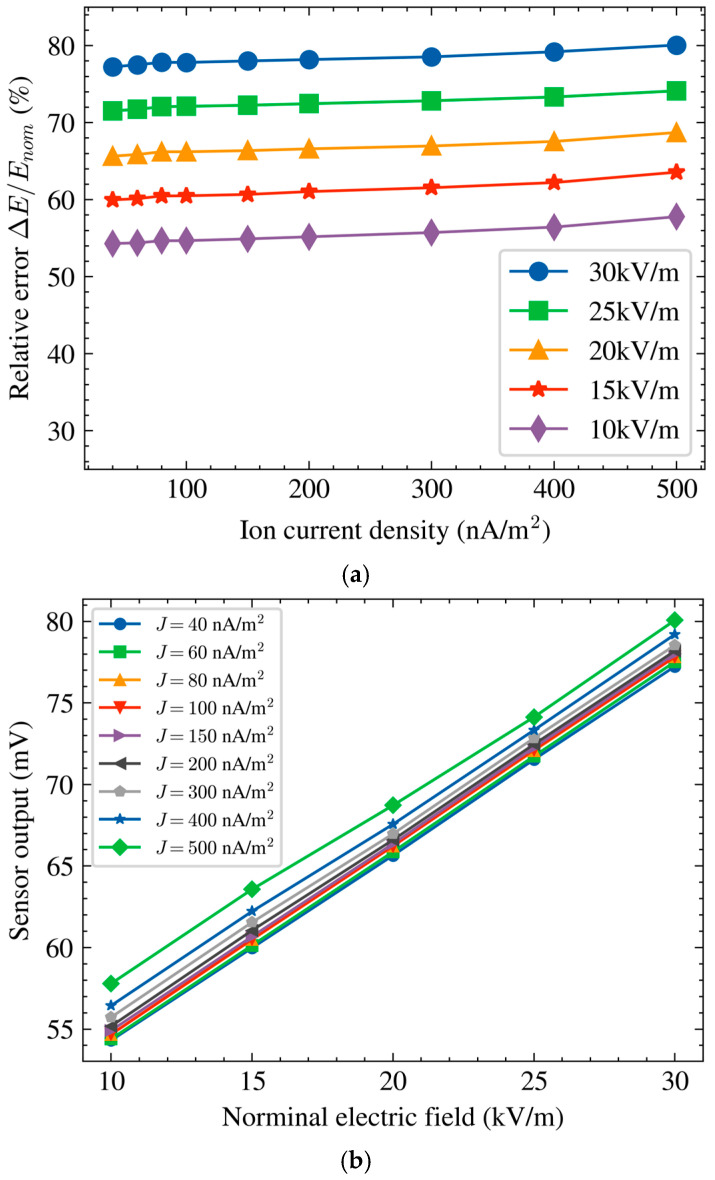
Sensor output characteristics under DC ionized field conditions: (**a**) sensor output voltage as a function of ion current density *J* at five nominal electric field levels; (**b**) sensor output voltage versus nominal electric field $E_{nom}$ under nine ion current density levels.

For the experiments in Figure 7, the measurement data were collected within a limited time interval after the experimental voltage settings were established. Although a slow drift exists under ionized conditions, the drift rate is relatively low within the measurement interval and does not significantly affect the calibration characteristics.

Figure 7a provides a direct view of the sensor output $V_{\mathrm{out}}$ (mV) as the ion current density J increases under five nominal electric field settings ($E_{\mathrm{nom}}$ = 10–30 kV/m). The five curves form distinct bands in output level, reflecting the different nominal field conditions, while all curves show a consistent upward trend with increasing J. This indicates that the output increases as the ion current density rises, even when the nominal field setting remains unchanged. Over the range of J  = 40–500 nA/m^2^, the output change within each curve reaches approximately 2.60–3.59 mV, which is sufficiently large to be visible in the raw data and implies that ion flow can induce a noticeable output shift.

To better understand the sensor response under varying J  and nominal electric field conditions, Figure 7b presents the output curves $V_{\mathrm{out}}$ versus $E_{\mathrm{nom}}$ at different ion current density levels. As J increases, the output curves are progressively separated and shifted upward. The separation among curves becomes more pronounced at higher J, which is consistent with the visual observation that stronger ion flow produces a larger departure from the electrostatic baseline behavior.

**Figure 8:**
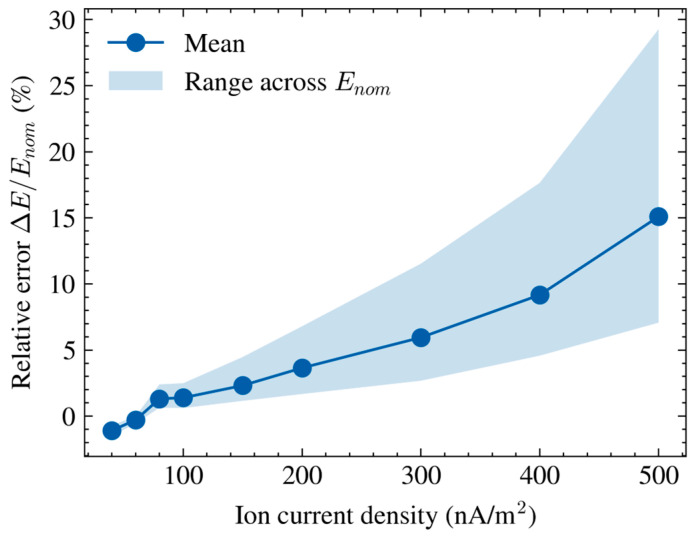
Relative error as a function of ion current density.

As shown in Figure 8, the relative error, defined as $\Delta E/E_{\mathrm{nom}}$, represents the difference between the field estimated using electrostatic calibration and the nominal field $E_{\mathrm{nom}}$. The mean relative error increases as ion current density J rises. The shaded band represents the error range across the five nominal electric field levels. The mean error increases from −1.09% at low J to 15.10% at high J, indicating that the deviation grows substantially as ion current density increases. Moreover, the error range broadens from −1.50% to −0.70% at low J to 7.07–29.28% at high J, indicating that the deviation becomes both larger and more variable across nominal electric field levels under stronger ion flow conditions.

This widening error range reflects the fact that the deviation is not uniform across all nominal field settings, which is particularly significant under stronger ion flow. At lower J values, the error remains relatively small and more consistent across the field settings, but as J increases, the curves diverge, revealing that ion flow has a compounded effect on the sensor output. This divergence of the curves shows that the deviation cannot be captured by a single constant correction term and further supports the need for a calibration approach that incorporates both ion current density and nominal electric field settings.

Notably, the maximum relative error observed in Figure 8 reaches 29.28% at high J, underscoring the importance of accounting for ion flow effects when performing calibration in DC ionized environments.

## 4. Calibration Method Incorporating Ion Current Density

Based on the observed output characteristics, the sensor response in DC ionized fields can be described by a joint dependence on the nominal electric field and ion current density. For a given ion flow condition, the output voltage of the MEMS electric field sensor remains approximately linear with respect to the nominal electric field. This behavior motivates the following calibration model:

(5) $$V_{out}(E_{nom},J)=S(J)E_{nom}+V_{0}(J)$$

where $V_{\mathrm{out}}$ is the sensor output voltage, S(J) represents the ion-density-dependent sensitivity, and $V_{0}(J)$ denotes the corresponding offset. The calibration problem becomes the identification of the two J-dependent parameters S(J) and $V_{0}(J)$, and their use for field reconstruction under arbitrary ion flow conditions within the calibrated range.

**Step 1: Parameter extraction at discrete ion flow conditions.**

Calibration experiments are conducted at a set of discrete ion current density levels $\left\{J_{i}\right\}$. For each $J_{i}$, the nominal electric field is swept over the predefined set $\left\{E_{\mathrm{nom},k}\right\}$, while the ion current density is monitored by the Wilson plate. The corresponding sensor output is recorded at each $\left(E_{\mathrm{nom},k},J_{i}\right)$. A linear regression of $V_{\mathrm{out}}$ versus $E_{\mathrm{nom}}$ is then performed at each $J_{i}$ to obtain the parameter pair $\left(S_{i},V_{0,i}\right)$. Repeating this procedure yields two discrete datasets $\left\{\left(J_{i},S_{i}\right)\right\}$ and $\left\{\left(J_{i},V_{0,i}\right)\right\}$.

**Step 2: Continuous mapping of calibration parameters.**

The parameter sets $\left\{\left(J_{i},S_{i}\right)\right\}$ and $\left\{\left(J_{i},V_{0,i}\right)\right\}$ are extended to continuous mappings S(J) and $V_{0}(J)$ by interpolation in the J-domain. A shape-preserving interpolation scheme is adopted to ensure smooth transitions between adjacent points and to avoid nonphysical oscillations. The resulting mappings enable evaluation of S and $V_{0}$ for a given ion current density J within the calibrated interval.

**Step 3: Calibration application.**

During measurements in DC ionized fields, the corresponding calibration parameters S(J) and $V_{0}(J)$ are obtained from the interpolated mappings, and the electric field is reconstructed according to the inverse form of (4):

(6) $$E_{\mathrm{est}}=\frac{V_{\mathrm{out}}-V_{0}(J)}{S(J)}$$

where $E_{\mathrm{est}}$ is the estimated electric field.

**Step 4: Validation protocol.**

To evaluate whether the J-dependent calibration can generalize across ion flow conditions, a leave-one-out validation is performed. Each $J_{i}$ is removed in turn, the remaining points are used to construct the parameter mappings, and the removed $S_{i}$ and $V_{0,i}$ are predicted. The predicted parameters are then used to reconstruct the field at the tested $E_{\mathrm{nom},k}$ points, and the resulting errors in S, $V_{0}$, and the reconstructed field are calculated as evaluation metrics.

## 5. Experiment Results and Discussion

**Figure 9:**
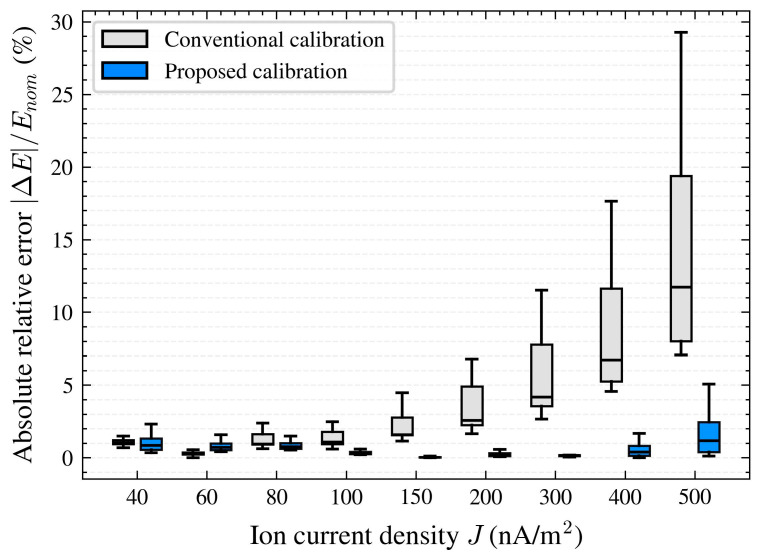
Absolute relative error before (conventional calibration) and after (proposed calibration) under different ion current density levels, calculated from five nominal electric field settings.

The effectiveness of the proposed calibration method is evaluated by comparing the sensor output error distributions before and after applying the ion-current-density-dependent calibration. Figure 9 presents the boxplot of absolute relative errors $|\Delta E|/E_{\mathrm{nom}}$ under different ion current density conditions for both the conventional electrostatic calibration and the proposed calibration method. Each boxplot is derived from five measurements corresponding to five nominal electric field values, ranging from $E_{\mathrm{nom}}=10$ kV/m to $E_{\mathrm{nom}}=30$ kV/m.

As illustrated in Figure 9, the boxplots provide a distribution-level comparison of the absolute relative error across ion current density conditions. Under the conventional calibration, the error distribution shifts upward and becomes progressively broader as ion current density increases, reflected by higher medians, larger interquartile ranges, and an extended upper whisker. This indicates that the deviation is not only larger on average but also more dispersed across the nominal field settings when ion flow becomes stronger. After applying the proposed calibration, the boxplots are consistently more compact across the ion current density range, with reduced interquartile ranges and a shortened upper tail, indicating a narrower error spread and a reduced occurrence of large deviations.

The numerical results in Table 1 present the detailed comparison of the minimum, maximum, and mean errors before and after applying the proposed calibration method for different ion current densities. The results demonstrate a significant reduction in error across ion current densities, with the maximum error dropping from a worst case of 29.28% to approximately 5.07% after calibration.

At the lower end of ion current density, the error statistics remain broadly comparable before and after calibration, and the mean error shows only limited change. In this regime, the ion-flow-related contribution to the sensor deviation is weaker, so the benefit of introducing an ion-current-density-dependent correction is naturally less pronounced; meanwhile, for the lower nominal-field points, the signal-to-noise ratio can be relatively lower and the ion-flow-induced perturbation may account for a larger fraction of the measured response. As a result, ratio-based error metrics can become less stable and may occasionally exhibit a slight increase for some conditions. As ion current density increases, the mean error is markedly reduced after calibration. For example, the mean error decreases from 9.17% to 0.62% at J=400 nA/m^2^ and from 15.10% to 1.85% at J=500 nA/m^2^, showing that the proposed method provides more pronounced improvement under stronger ion flow conditions.

## 6. Conclusions

In this study, a calibration method for MEMS electric field sensors in DC ionized environments is proposed by incorporating ion current density into the calibration process. The experimental results show that, compared with conventional electrostatic calibration, the proposed method reduces measurement errors, with the improvement being more evident at higher ion current densities. The maximum relative error is substantially reduced.

The proposed calibration approach accounts for the influence of ion flow on the sensor response. Under lower ion current density conditions, the method remains stable with only small variations in error. At higher ion current densities, the error reduction becomes more pronounced, indicating the necessity of incorporating ion current density to compensate for the increasing deviation under stronger ion flow.

This work provides a calibration methodology for applying MEMS electric field sensors in DC ionized environments, relevant to high-voltage monitoring and related measurement tasks. Future research will focus on validating this method under additional operational conditions and expanding its application to other sensor types and measurement scenarios.

## Figures and Tables

**Table 1:** Summary of absolute relative errors $|\Delta E|/E_{\mathrm{nom}}$ for each ion current density condition before and after applying the proposed calibration.

J (nA/m^2^)	Min Error Before (%)	Min Error After (%)	Max Error Before (%)	Max Error After (%)	Mean Error Before (%)	Mean Error After (%)
40	0.6964	0.3607	1.5033	2.3284	1.0859	1.0887
60	0.0072	0.4209	0.5557	1.5829	0.2876	0.8508
80	0.6398	0.5285	2.4049	1.4925	1.3107	0.8852
100	0.6111	0.2125	2.4910	0.6147	1.3911	0.3613
150	1.1567	0.0083	4.4724	0.1167	2.3175	0.0483
200	1.6736	0.0808	6.7983	0.5885	3.6470	0.2687
300	2.6786	0.0667	11.5364	0.2026	5.9505	0.1524
400	4.5738	0.0201	17.6527	1.6798	9.1684	0.6169
500	7.0721	0.1242	29.2825	5.0703	15.0952	1.8475

## Data Availability

The original contributions presented in the study are included in the article. Further inquiries can be directed to the corresponding author.
